# Flagella Overexpression Attenuates *Salmonella* Pathogenesis

**DOI:** 10.1371/journal.pone.0046828

**Published:** 2012-10-03

**Authors:** Xinghong Yang, Theresa Thornburg, Zhiyong Suo, SangMu Jun, Amanda Robison, Jinquan Li, Timothy Lim, Ling Cao, Teri Hoyt, Recep Avci, David W. Pascual

**Affiliations:** 1 Department of Immunology & Infectious Diseases, Montana State University, Bozeman, Montana, United States of America; 2 Imaging and Chemical Analysis Laboratory, Department of Physics, Montana State University, Bozeman, Montana, United States of America; 3 Department of Infectious Diseases and Pathology, University of Florida, Gainesville, Florida, United States of America; The Scripps Research Institute and Sorrento Therapeutics, Inc., United States of America

## Abstract

Flagella are cell surface appendages involved in a number of bacterial behaviors, such as motility, biofilm formation, and chemotaxis. Despite these important functions, flagella can pose a liability to a bacterium when serving as potent immunogens resulting in the stimulation of the innate and adaptive immune systems. Previous work showing appendage overexpression, referred to as attenuating gene expression (AGE), was found to enfeeble wild-type *Salmonella*. Thus, this approach was adapted to discern whether flagella overexpression could induce similar attenuation. To test its feasibility, flagellar filament subunit FliC and flagellar regulon master regulator FlhDC were overexpressed in *Salmonella enterica* serovar Typhimurium wild-type strain H71. The results show that the expression of either FliC or FlhDC alone, and co-expression of the two, significantly attenuates *Salmonella*. The flagellated bacilli were unable to replicate within macrophages and thus were not lethal to mice. In-depth investigation suggests that flagellum-mediated AGE was due to the disruptive effects of flagella on the bacterial membrane, resulting in heightened susceptibilities to hydrogen peroxide and bile. Furthermore, flagellum-attenuated *Salmonella* elicited elevated immune responses to *Salmonella* presumably via FliC’s adjuvant effect and conferred robust protection against wild-type *Salmonella* challenge.

## Introduction

*Salmonella enterica* are Gram-negative pathogens capable of infecting humans and animals causing salmonellosis [1]. Although more than 2,500 *S. enterica* serovars have been identified to date [2], only a few cause the majority of infections [3]. *S. enterica* serovar Typhi causes typhoid fever, which annually accounts for 16 million cases worldwide [4], while serovar Typhimurium causes ∼1.3 billion cases of non-typhoid fever globally each year [1], [5]. The most common *S*. Typhimurium isolated from humans is definitive phage type DT104, which has acquired multiple drug resistance [6]. DT104 is widely distributed in food animals and has been implicated in increased morbidity and mortality when compared with pan-susceptible *Salmonella* [7]. Therefore, a protective and cost-effective vaccine against typhoid and non-typhoid fevers would constitute an important control measure.

Currently, two typhoid vaccines are commercially available: attenuated *S.* Typhi strain Ty21a and the purified capsular polysaccharide of *S.* Typhi antigen Vi [8], [9]. Live oral vaccine Ty21a is well-tolerated, but modestly immunogenic, requiring 3–4 consecutive doses to achieve moderate levels of protection [10], [11]. Intramuscular vaccine Vi is protective but commonly associated with injection site reactions [12]. Thus, although Ty21a is licensed in 56 countries and Vi is licensed in more than 92 countries [13], neither has been widely adopted in public health programs in countries where typhoid and non-typhoid fevers are endemic [8], [9]. During the past few decades, efforts have been devoted to define virulence gene interruption to develop live *Salmonella* vaccines. However, none of these mutants is yet licensed for human application, which implies the conventional method for attenuating wild-type (wt) *Salmonella* to generate a live vaccine remains problematic. Therefore, the quest for new strategies to attenuate bacterial pathogens may expedite the development of *Salmonella* vaccines.

To enable pathogenesis, *Salmonella* has an array of specific virulence genes for expression at different stages of infection [14]. Of note is flagella-mediated virulence: on one hand, flagella increase the invasiveness of salmonellae [15]; and on the other hand, flagellin monomers induce an elevated innate immune response [16], [17] to incur bacterial clearance from the host. We questioned whether the latter property could be used to inactivate *Salmonella* pathogenesis. Although flagella are involved in various important functions, including biofilm formation [18], symbiosis [19], wetness sensing [20], mechanosensing [21], and interspecies communication [22], these are not constitutively expressed. Their expression occurs in response to stimuli, such as wetness [20], temperature, phase, and the viscosity and osmolarity of the medium [23], and is normally down-regulated inside the host [24]. The expression of flagella is very likely tightly regulated to prevent flagella-associated vulnerabilities from being exposed to the host. To test the hypothesis that heterologous or attenuating gene expression (AGE) can be used to enfeeble *Salmonella* [25], flagella were overexpressed in *S*. Typhimurium. The results show that flagella overexpression dramatically attenuates *Salmonella* virulence both *in vitro* and *in vivo* and is capable of conferring protection against salmonellosis.

## Results

### Overexpression of Flagella Inhibits *Salmonella* Growth

**Figure 1:**
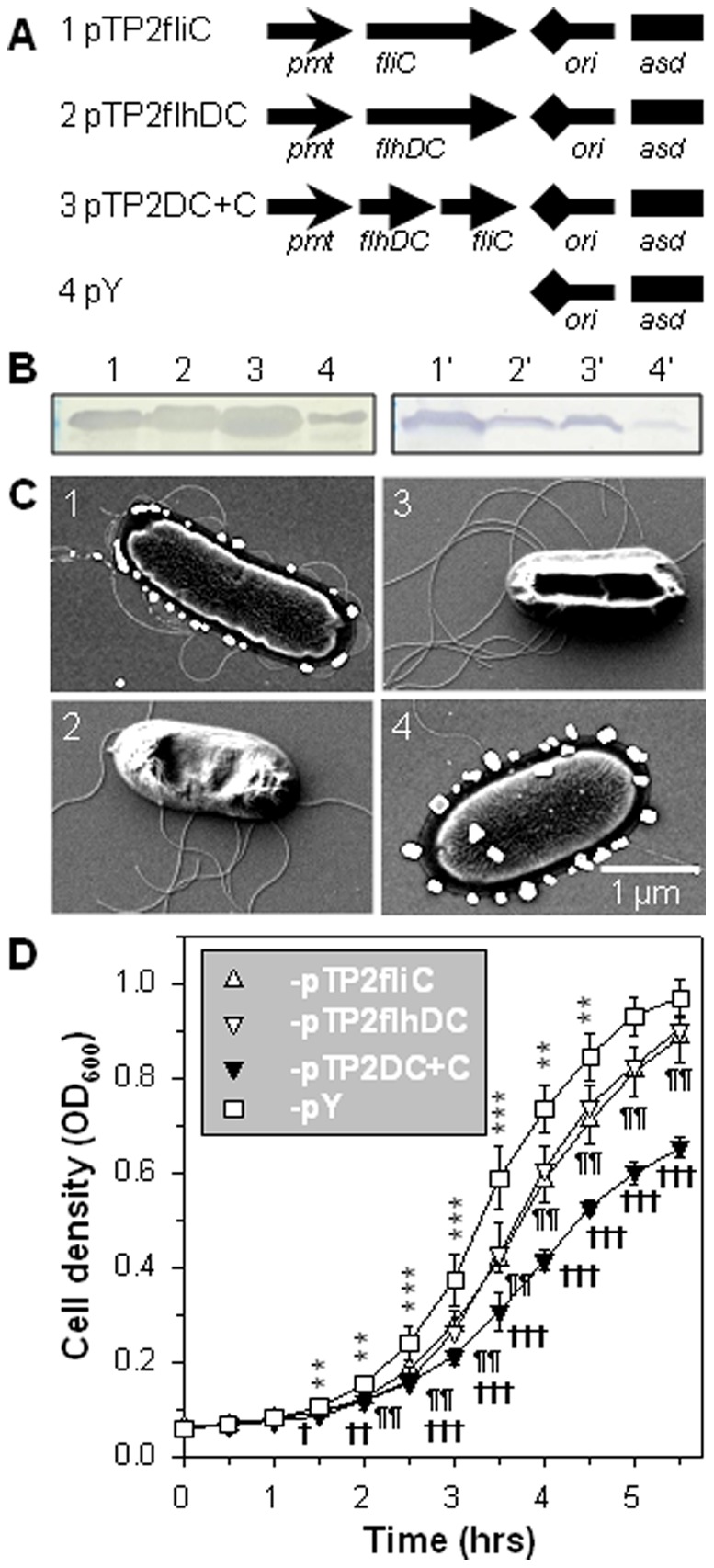
Characterization of flagellated *Salmonella* strains. (**A**) Schematic maps of plasmids (1) pTP2fliC, (2) pTP2flhDC, (3) pTP2DC+C, and (4) pY. The term “*pmt*” indicates fusion promoter of P*tetA*∼P*phoP*. (**B**) Detection of flagellin expression via Western blot. (1–4) The extracellular flagella sheared from cell surfaces were detected. (1′-4′) The intracellular flagellin contained within bacterial cells was detected. Approximately 7.2×10^8^ CFU of each strain were loaded into SDS-PAGE wells. (**C**) Flagellum observation via FESEM. Cells depicted for each strain possessed approximately the average number of flagellum filaments for that strain. (**D**) Overexpression of flagella hinders bacterial growth. Recombinant *Salmonella* strains were analyzed for growth rate indexed by OD_600_ value, and the statistical differences were indicated: **P<0.01 and ***P<0.001 for P1-pTP2fliC vs. -pY; ^¶¶^ *P*<0.01 for -pTP2flhDC vs. -pY; and ^†^ *P*<0.05, ^††^ *P*<0.01, and ^†††^ *P*<0.001 for -pTP2DC+C vs. -pY. Depicted are the mean of triplicate samples ± SEM (n = 3 experiments).

Plasmids pTP2fliC, pTP2flhDC, and pTP2DC+C were constructed using *E. coli* H681 (Figure 1A). They were transformed to *Δasd S*. Typhimurium P1 [25] to obtain three new strains: P1-pTP2fliC, -pTP2flhDC, and -pTP2DC+C. The previously constructed strain P1-pY, which recovers full virulence to its parental strain H71 [25], was used as a control. To assess the expression levels of FliC by each construct, a Western blot analysis was performed to measure sheared and cell-associated FliC. Depicted in ascending order (Figure 1B), P1-pTP2fliC, -pTP2flhDC, and -pTP2DC+C showed enhanced FliC expression relative to -pY strain. FESEM analysis confirmed the Western blot results. P1-pTP2DC+C cells were entangled with dense flagella, -pTP2flhDC and -pTP2fliC cells showed less, and -pY cells exhibited sparse flagella (Figure 1C). These results show that the overexpression of either *fliC* or *flhDC* alone enhances flagellum expression relative to P1-pY, whereas co-expression of *fliC* and *flhDC* further promotes greater flagella expression.

The growth rates of the four strains, P1-pTP2fliC, -pTP2flhDC, -pTP2DC+C, and -pY, were then compared in LB at 37°C. As early as 1.5 hrs post-inoculation, P1-pTP2fliC and -pTP2DC+C grew significantly more slowly than -pY, and -pTP2flhDC grew significantly more slowly than -pY at two hrs post-inoculation (Figure 1D). In the logarithmic growth phase, P1-pTP2fliC, -pTP2flhDC, and -pTP2DC+C displayed slower growth rates than -pY. The strain expressing *fliC* alone, P1-pTP2fliC, had a growth rate similar to that of the strain expressing *flhDC* alone, -pTP2flhDC, and both grew faster than -pTP2DC+C. Hence, this result suggests that the overexpression of *fliC*, *flhDC*, or both represses bacterial growth.

### Overexpression of Flagella Inactivates *Salmonella* Viability

**Figure 2:**
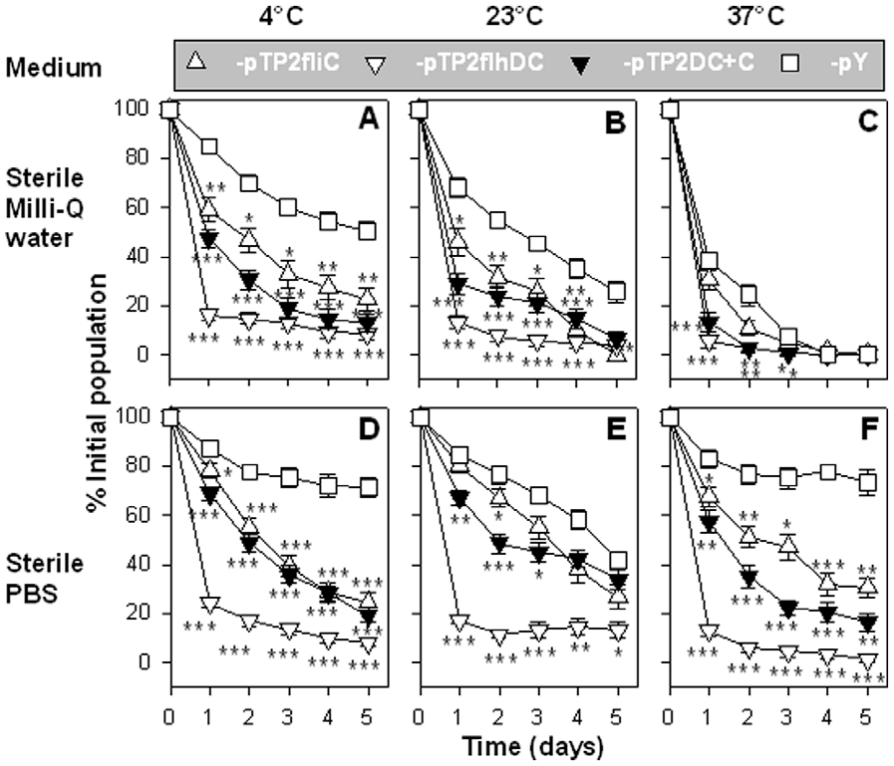
Overexpression of flagella reduces *Salmonella* viability. Recombinant *Salmonella* strains were analyzed for *ex vivo* viability in Milli-Q water (**A**, **B**, **C**) and sPBS (**D**, **E**, **F**) at 4°C (**A**, **D**), 23°C (**B**, **E**), and 37°C (**C**, **F**). The percentages of live cells were analyzed on a daily base. The statistical differences were calculated to be *P<0.05, **P<0.01, and ***P<0.001 for P1-pTP2fliC, -pTP2flhDC, or -pTP2DC+C vs. -pY. Depicted are the mean ± SEM (n = 5 experiments and 6 samples/experiment).

To determine whether the overexpressed flagella produced any impact on the viability of the salmonellae, a viability assay was conducted. The results showed that at nearly all sampling time points, regardless of temperature (4°C, 23°C, or 37°C) or medium (Milli-Q water or sterile phosphate-buffered saline (sPBS)), the survival rates of P1-pTP2fliC, -pTP2flhDC, and -pTP2DC+C were significantly less than that of control -pY (Figure 2). In general, the survival rates of all tested strains were diminished in Milli-Q water, although this was not always the case when they were in sPBS. The harshest condition for all tested strains was Milli-Q water at 37°C, in which cells from all strains died quickly (Figure 2C). All strains survived better in sPBS than in Milli-Q water. This was particularly true when the temperature was 23°C or 37°C. This indicates that a balanced osmotic condition is favorable for maintaining the salmonellae viability. Overall, overexpression of flagella results in the decreased viability of salmonellae *ex vivo*.

### Overexpression of Flagella Attenuates *Salmonella* Virulence

**Figure 3:**
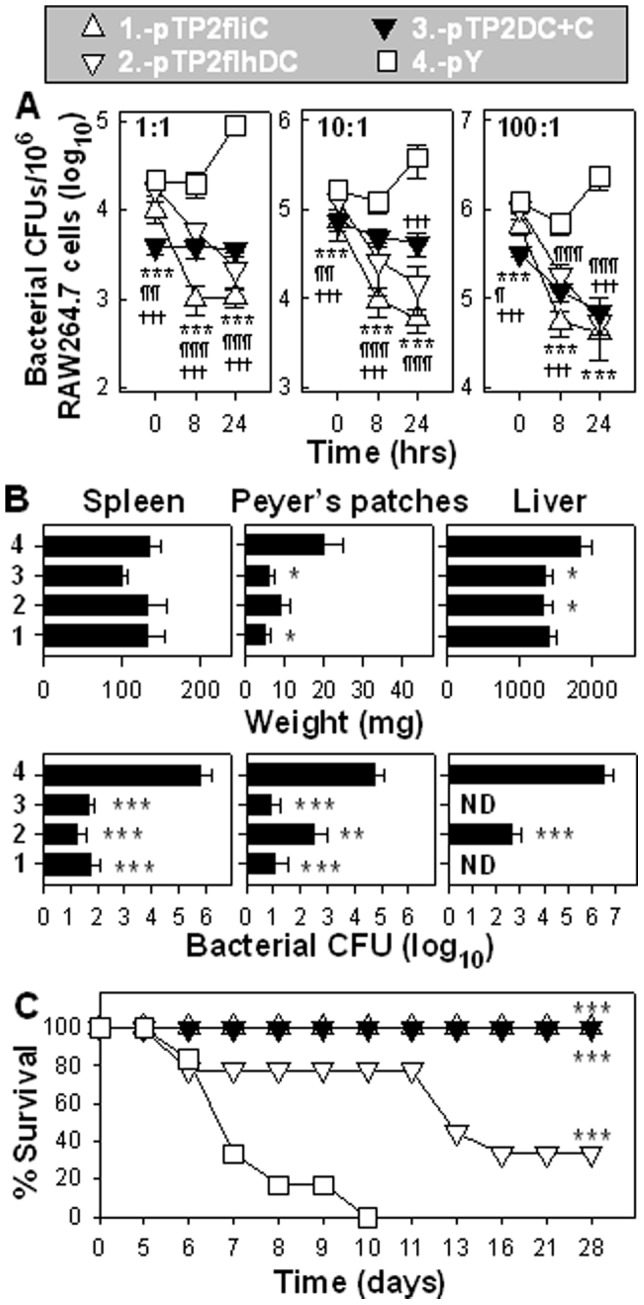
Overexpression of flagella attenuates *Salmonella* virulence. (**A**) Recombinant *Salmonella* strains were analyzed for virulence in RAW264.7 macrophages. At all of the three bacteria-to-macrophage infection ratios of 1∶1, 10∶1, and 100∶1, P1-pTP2fliC, -pTP2flhDC, and -pTP2DC+C were not able to reproduce in macrophages, while -pY did. The statistical differences in bacterial CFUs were calculated to be ***P<0.001 for P1-pTP2fliC vs. -pY; ^¶^ *P*<0.05, ^¶¶^ *P*<0.01, and ^¶¶¶^ *P*<0.001 for -pTP2flhDC vs. -pY; and ^†††^ *P*<0.001 for -pTP2DC+C vs. -pY. Depicted are the mean ± SD (n = 3 independent experiments). (**B**) Recombinant *Salmonella* strains were evaluated for ability to colonize spleen, Peyer’s patches, and liver 4 days after oral infection. The statistical differences in tissue weights and CFUs were calculated to be *P<0.05, **P<0.01, and ***P<0.001 for P1-pTP2fliC, -pTP2flhDC, or -pTP2DC+C vs. -pY; ND: not detectible. A total of five to seven mice were used in each group, and the depicted results are the mean ± SEM. (**C**) Survival fractions of the mice orally infected with 1×10^9^ CFUs of P1-pTP2fliC, -pTP2flhDC, or -pTP2DC+C were compared with that of -pY, ***P<0.001. A total of nine mice were used for P1-pTP2fliC, -pTP2flhDC, or -pTP2DC+C, with six mice were used for -pY. Depicted are the mean of two independent experiments.

To assess their susceptibility to killing by macrophages, these four strains were tested for survival in RAW264.7 cells. At every infection ratio, P1-pTP2fliC, -pTP2flhDC, and -pTP2DC+C were less capable of infecting macrophages than -pY shortly after onset of infection (t = 0 hr) (Figure 3A). After infection, P1-pTP2fliC, -pTP2flhDC, and -pTP2DC+C were unable to survive in macrophages, regardless of infection dose, as their bacterial burdens were diminished at 8 and 24 hrs post-infection. In contrast, at all of the tested infection doses, control P1-pY was able to reproduce within macrophages, since its bacterial colony forming units (CFUs) at 24 hrs were greater than its initial CFUs (t = 0). This is similar to what we observed previously [25]. These results suggest that overexpression of flagella limits the recombinant *Salmonella’s* capacity for infection, survival, and multiplication within macrophages.

Since the genetic manipulations were performed in wt *Salmonella*, we queried whether these recombinant strains would be attenuated *in vivo*. Groups of mice were orally infected with one of each strain, and four days later, spleens, Peyer’s patches, and livers were evaluated for extent of colonization. No differences were discerned for the splenic weights among the four groups, but significant differences were found in the Peyer’s patches and liver weights between P1-pTP2fliC, -pTP2flhDC, or -pTP2DC+C, and -pY (*P*<0.01). The splenic bacterial burdens for P1-pTP2fliC, -pTP2flhDC, and -pTP2DC+C were 11,002-, 41,256-, and 13,752-fold, respectively, less than those of -pY (Figure 3B). In the Peyer’s patches, the bacterial burdens for P1-pTP2fliC, -pTP2flhDC, and -pTP2DC+C were 4,612-, 179-, and 6,918-fold, respectively, less than those of -pY. In livers, the P1-pTP2flhDC load was 6,370-fold less than -pY-infected mice, while no salmonellae were detected in -pTP2fliC- or -pTP2DC+C-infected mice. These findings explicitly show that overexpression of flagella attenuates *Salmonella*’s virulence *in vivo* and thus results in *Salmonella* being less inflammatory to the host.

To assess whether these recombinant strains are lethal, groups of mice were orally infected and monitored for 4 weeks. All mice administered P1-pTP2fliC or -pTP2DC+C survived (9/9 and 9/9, respectively), one third of mice administered -pTP2flhDC survived (3/9), but all mice dosed with -pY succumbed to infection (6/6) (Figure 3C). Lethality to mice seems to be associated with liver colonization, since both P1-pTP2flhDC and -pY are able to colonize liver (Figure 3B), and mice from these two groups died. In contrast, P1-pTP2fliC and -pTP2DC+C are unable to colonize the liver, and all mice in these two groups survived. This result further indicates that the overexpression of flagella greatly attenuates *Salmonella in vivo* and that the expression of main filament subunit FliC alone is able to vitiate the lethal capacity of *Salmonella*.

### Mechanism of Flagellum-mediated *Salmonella* Attenuation

**Figure 4:**
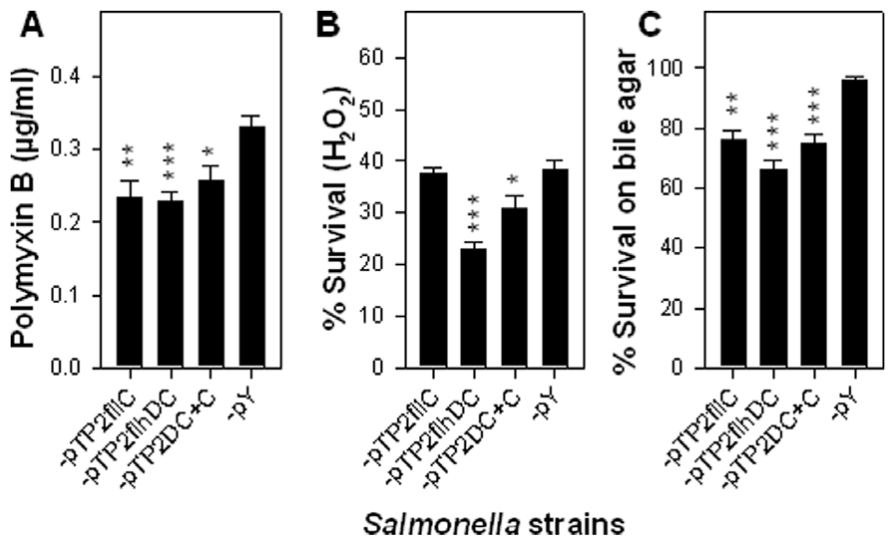
Flagellated *Salmonella* strains show enhanced sensitivity to treatments with polymyxin B (PMB), hydrogen peroxide, and bile salt. The sensitivities of the flagellated *Salmonella* strains to (**A**) PMB, (**B**) H_2_O_2_, and (**C**) bile salt were determined. The PMB MIC, the survival rates after H_2_O_2_ treatment or in the presence of bile salt were statistically calculated and are indicated: *P<0.05, **P<0.01, and ***P<0.001 for P1-pTP2fliC, -pTP2flhDC, or -pTP2DC+C vs. -pY. Depicted are the mean ± SEM (n = 3).

To understand the mechanism by which overexpression of flagella causes *Salmonella* attenuation, the flagellated salmonellae were subjected to antimicrobial assays (Figure 4). Polymyxin B (PMB), a peptide antibiotic, is lethal to gram-negative bacteria via binding lipid A [26]. To test whether the flagellated strains exhibit an increased susceptibility to PMB, we measured the minimum inhibitory concentration (MIC) of PMB for these four strains [25]. The results showed that the PMB MICs for P1-pTP2fliC, -pTP2flhDC, and -pTP2DC+C were all significantly less than that of control -pY (Figure 4A). Because PMB exerts elevated detrimental effects on bacteria with compromised outer membranes [27], this result implies that the cell membranes of flagellated salmonellae are permeabilized relative to control, P1-pY. Expectedly, the survival rates of strains P1-pTP2flhDC and -pTP2DC+C were significantly lower than that of -pY after cells were treated with hydrogen peroxide (Figure 4B), and the survival rates of -pTP2fliC, -pTP2flhDC, and -pTP2DC+C were all significantly less than that of -pY when grown on LB plus 1% bile salt (Figure 4C). These results suggest that overexpression of flagella disrupts the salmonellae membrane integrity, making them more susceptible to antimicrobial agents, i.e., hydrogen peroxide and bile. Altering their barrier function might be the reason these strains exhibited reduced viability *ex vivo* (Figure 2), reduced virulence *in vitro* (Figure 3A), and diminished virulence *in vivo* (Figure 3B and C).

### The Flagellum-attenuated *Salmonella* Confer Protection against wt *Salmonella* Challenge

**Figure 5:**
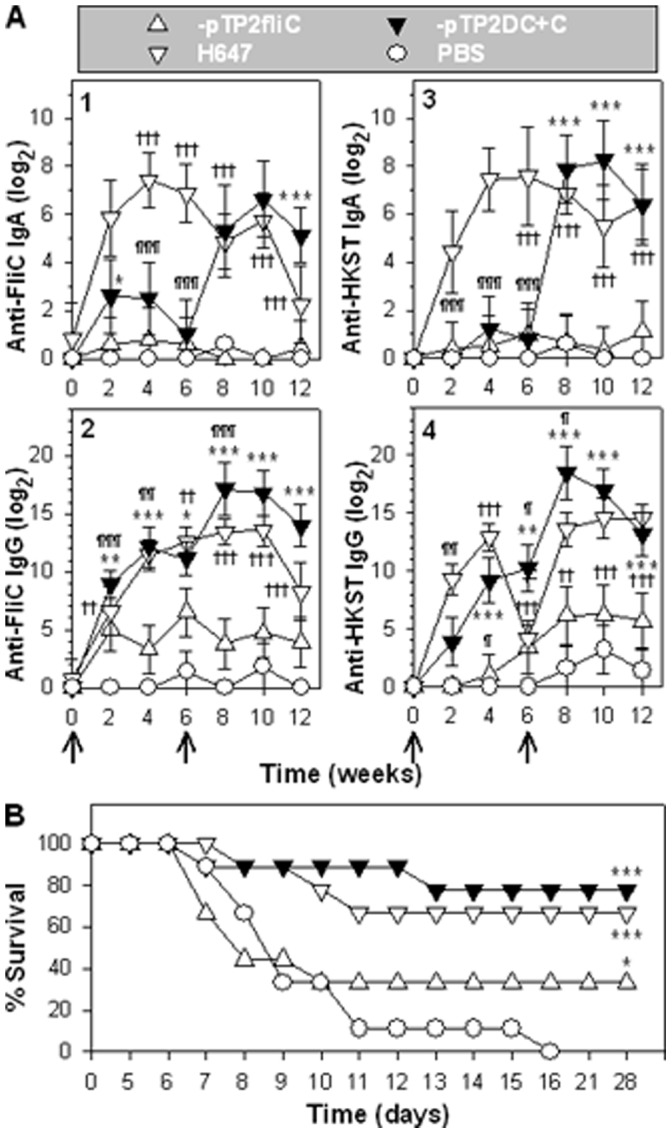
The flagellum-attenuated *Salmonella* strains confer protective immunity against wt *Salmonella* challenge. (**A**) ELISA titration of anti-FliC and anti-HKST Ab endpoint titers. BALB/c mice (4–5 individuals/group) were orally immunized with 1×10^9^ CFU of P1-pTP2fliC, -pTP2DC+C, H647, and sPBS at weeks 0 and 6. The copro-IgA (1, 3) and serum IgG (2, 4) specific for FliC (1, 2) and HKST (3, 4) Ab titers were measured biweekly, and the significant differences are indicated: *P<0.05, **P<0.01, and ***P<0.001 for P1-pTP2DC+C vs. -pTP2fliC; ^¶^ *P*<0.05, ^¶¶^ *P*<0.01, and ^¶¶¶^ *P*<0.001 for -pTP2DC+C vs. H647; and ^†^ *P*<0.05, ^††^ *P*<0.01, and ^†††^ *P*<0.001 for -pTP2fliC vs. H647. Each experiment was repeated twice, and depicted are the means ± SEM. (**B**) Protective efficacy assay. Immunized mice (**A**) were orally challenged with 5×10^7^ CFUs of wt *S*. Typhimurium H71, and survival fractions obtained from P1-pTP2fliC (n = 9 mice), -pTP2DC+C (n = 9 mice), or H647-dosed mice (n = 9 mice) were compared with sPBS-dosed (n = 9 mice) mice and significances are indicated: ***P<0.001 for P1-pTP2DC+C, -pTP2fliC, or H647 vs. sPBS. The depicted are the mean of two independent experiments.

Since P1-pTP2fliC and -pTP2DC+C are not lethal, we queried whether these strains could be used as vaccines for *Salmonella*. Groups of mice were vaccinated with P1-pTP2fliC, -pTP2DC+C, *ΔaroA S*. Typhimurium (strain H647), or sPBS. Mice were monitored biweekly for anti-FliC and anti-HKST (heat-killed *Salmonella* Typhimurium) antibody (Ab) responses. At 6 weeks post-primary immunization, mice were boosted with an additional oral dose. The vaccine strain H647-dosed mice [25] was used as a positive vaccination control, and the sPBS-dosed mice provided the negative control group. P1-pTP2DC+C induced stronger anti-FliC copro-IgA titers than those from -pTP2fliC-vaccinated mice, but less than H647 during the primary immunization phase (Figure 5A-1). After boosting, P1-pTP2DC+C induced much stronger anti-FliC titers than those of -pTP2fliC and H647. P1-pTP2DC+C and H647 induced greater anti-FliC serum IgG titers than -pTP2fliC, and after boost immunization, it showed the greatest serum IgG titers than any of the groups (Figure 5A-2). P1-pTP2DC+C and -pTP2fliC induced weaker anti-HKST copro-IgA titers than H647 during the priming phase (Figure 5A-3). After boosting, P1-pTP2DC+C induced similar titers to H647, but greater than -pTP2fliC. P1-pTP2DC+C and H647 induced similar anti-HKST serum IgG titers, but greater than -pTP2fliC during the priming phase (Figure 5A-4). Likewise after boosting, P1-pTP2DC+C induced similar anti-HKST titers to H647, but greater than -pTP2fliC. These results suggest that booster immunization is essential for P1-pTP2DC+C to elicit robust immune Ab titers. P1-pTP2DC+C is more immunogenic than -pTP2fliC regarding both the HKST and the flagellin antigens. These results suggest that flagella overexpression enhances Ab responses to FliC and HKST.

To evaluate its efficacy against wt strain challenge, these mice were subsequently orally challenged with wt *S*. Typhimurium H71 (Figure 5B). While the sPBS-dosed mice all succumbed to infection, mice vaccinated with P1-pTP2DC+C conferred 77.8% (7/9), H647 66.7% (6/9), and -pTP2fliC 33.3% (3/9) protection. These results show that P1-pTP2DC+C conferred the best protection against wt *Salmonella* infection.

## Discussion

In addition to being a virulence factor [28], flagella are involved in biofilm formation [18], accelerate bacterial invasion of host cells [29], are essential for multiplication *in vivo* [30], confer an advantage in the early stage of infection in animals [31], and activate the host innate immune system while inactivating apoptosis of epithelial cells [32]. Flagella also exhibit adjuvant properties acting as a TLR5 agonist [33], which render this structure a protective antigen against salmonellosis [34]. Hence, the exquisite regulation of flagella is crucial for *Salmonella* when interacting with the animal host. Flagellum expression has the potential to turn on to provide a great advantage in the early stage of infection [31], and then turn off to minimize host recognition once infection can be established [24]. Consequently, *Salmonella* tightly controls its flagellum expression.

Since bacterial self-preservation relies on the precise regulation of flagella, the absence of control of flagellum expression has formidable consequences. First, the constitutive expression of *flhDC* imposes a heavy metabolic burden on the cells as previously observed [35], which is evidenced by the slow growth rates of P1-pTP2flhDC versus (vs.) -pY (Figure 1D). This is because a number of pathways or genes are under the control of FlhDC [22], and as a result, their continued “on” status consumes much of the substrates and energy. This is further supported by the observation that co-expression of *fliC* and *flhDC* in strain P1-pTP2DC+C results in an elevated flagellin yield impeding cell growth (Figure 1D). Second, the constitutive expression of *flhDC* causes salmonellae to be unable to survive in water or sPBS, since the population of P1-pTP2flhDC abates more rapidly when compared to the virulence-restored control strain, -pY (Figure 2). In contrast, co-expression of *flhDC* and *fliC* in strain P1-pTP2DC+C improves survival relative to -pTP2flhDC. The reason for this outcome is not immediately clear. However, since P1-pTP2DC+C produces more flagellin than -pTP2flhDC, we may deduce that FliC has some protective effects on the -pTP2flhDC-damaged membrane. Although there were no significant differences in their sensitivities to PMB, significant differences in their sensitivities to hydrogen peroxide treatment were observed as evidenced by the survival rates for P1-pTP2DC+C and -pTP2flhDC being 31.0% vs. 23.0% (*P*<0.01), respectively, and when grown on LB agar plus 1% bile salt, the survival rates for -pTP2DC+C and -pTP2flhDC were 74.8% vs. 65.9% (*P*<0.05). These differences in susceptibilities to antimicrobials suggest FliC may confer some protection. Consequently, P1-pTP2flhDC and -pTP2DC+C are greatly attenuated as analyzed *in vitro* and *in vivo*. Third, both strains' capabilities of infecting, surviving, and replicating within the macrophages are diminished when compared to the virulence-restored control strain, P1-pY. Likewise, their abilities to colonize mouse tissues are also compromised (Figure 3). However, P1-pTP2DC+C is nonlethal to mice, while -pTP2flhDC still retains some virulence implicating FliC has a role in this attenuation.

FliC expression alone is sufficient to attenuate wt *Salmonella*. The overexpression of *fliC* in P1-pTP2fliC imposes growth restrictions, diminishes viability in water and sPBS, reduces its capacity to infect and replicate in macrophages, and reduces its capacity to colonize mouse tissues relative to control -pY strain. In fact, all mice dosed with P1-pTP2fliC survived (Figure 3C). It seems that the AGE effects of FliC differ from FlhDC, which relates to the milieu they encounter. When evaluated *ex vivo*, P1-pTP2fliC had greater viability in both water and sPBS than -pTP2flhDC. When evaluated during infection, P1-pTP2fliC was less viable than -pTP2flhDC as evidenced by the fewer CFUs at 8 and 24 hrs post-infection of macrophages. *In vivo*, P1-pTP2fliC was less virulent than -pTP2flhDC and may be linked to the former inability to colonize the liver while the latter can. Although both of these strains appeared attenuated, at least by the criterion in RAW264.7 macrophages, unlike P1-pTP2fliC, -pTP2flhDC was lethal to mice. The fact that P1-pTP2flhDC remained lethal to mice suggests that this strain may regain its virulence *in vivo*. This restored virulence may be attributed to enhanced degradation of FlhDC *in vivo*, resulting in the salmonellae recovering part of their virulence. In fact, the rapid degradation of FlhDC has been previously observed in *Proteus mirabilis* [36].

Constitutive to its antimicrobial defense, host phagocytic cells produce hydrogen peroxide to defend against bacterial infections [37]. Secreted bile provides another antimicrobial barrier interfering with the bacterial membrane and being bactericidal [38]. The increased susceptibilities by the flagellated *Salmonella* strains to antimicrobial attack may account for the underlying mechanisms that these strains are attenuated.

Attenuation of wt *Salmonella* via AGE rather than the virulence gene deletion has been previously demonstrated to be a useful method [25], [39]. AGE-impaired strains have been found to elicit robust immune responses against *Salmonella* and confer substantial protection [25]. In this study, we further showed that overexpression of a homologous antigen, FliC, is also capable of attenuating wt *Salmonella* to generate live vaccine, e.g., P1-pTP2DC+C, which provided the best protection against wt *Salmonella* challenge. Hence, using AGE as a method described in this study demonstrates one alternative to impair *Salmonella* and other Gram-negative bacteria as an approach to generate live bacterial vaccines.

## Materials and Methods

### Ethics Statement

All animal care and procedures were in accordance with institutional policies for animal health and well-being and approved by Montana State University Institutional Animal Care and Use Committee under protocol 2009-30. After challenge, mice were observed for symptoms twice daily for 4 weeks. An alternate source of water such as a sterile water gel was provided if they showed retardation in reaching the water. However, if they became moribund (difficult to move to reach food and water, and combined with serious fur ruffling) they were euthanized by CO_2_ and recorded as death.

### Bacterial Strains, Media, Plasmids, Primers and Growth Conditions

*Δasd Salmonella* Typhimurium strain P1 (Table 1) was transformed with *asd* ^+^ plasmids carrying heterologous genes, and the recombinant strains were stocked at −80°C. Bacterial organisms in the logarithmic growth phase were harvested from liquid lysogeny broth (LB) medium, and the cell optical density at 600 nm (OD_600_) was adjusted to ∼0.05 with LB medium for growth rate analysis using BioScreen C at a continuous agitation of 150 rpm at 37°C for 5.5 hrs [39]. The growth rate of each strain was measured in triplicate per experiment, and each experiment was repeated three times. The susceptibilities of the recombinant *Salmonella* to hydrogen peroxide (2.5 mM) and bile salt (1%) were determined according to a protocol previously described [39]. The MIC of PMB was analyzed as previously described [25]. The logarithmic growth phase cells were used for field emission scanning electron microscopy (FESEM) observation for detection of flagellum expression [40]. The salmonellae were harvested after overnight growth on LB agar and resuspended in sPBS for oral immunizations, and morphological evaluations via FESEM. For each strain, 20 cells were imaged, and a representative example with the average amount of flagella is depicted.

**Table 1:** Bacterial strains, plasmids, and primers used in this study.

Strains	Characteristics	Sources
*E. coli* H681	Δ*asd*	[49]
*S*. Typhimurium H71	Wild-type strain	[49]
*S*. Typhimurium P1	Δ*asd*::*kan* ^R^ H71	[25], [50]
**Plasmids**	**Characteristics and derivation**	**Sources**
pV55	*asd* ^+^, *lcrV* under control of P*tetA*∼P*phoP*	[43]
pJGX15C-asd+	*asd* ^+^, *cfa*/I under control of P*tetA*	[49]
pTP2fliC	*asd* ^+^, *fliC* under control of P*tetA*∼P*phoP*	This study
pTP2flhDC	*asd* ^+^, *flhDC* under control of P*tetA*∼P*phoP*	This study
pTP2DC+C	*asd* ^+^, both *flhDC* and *fliC* under control of P*tetA*∼P*phoP*, derived from pTP2flhDC	This study
pY	*asd* ^+^, derived from pJGX15C-*asd* ^+^	[25]
**Primers**	**Oligonucleotide sequences^1^**	**Enzyme sites**
fliC-F	**GATATCGAGCTC** GGAGGAAAAGATCATGGCACAAG	*Eco*RI, *Sac*I
fliC-R	**CTCGAG** GCTCCGGAATTAAAAAAGG	*Xho*I
flhDC-F	**GAGCTC** GGAGGTTATTCTGGATGGGAACA	*Sac*I
flhDC-R	**GATATC** AAGCTTACCGCTGCTGGAGTG	*Eco*RI

Note: ^1^The sequences in bold print are the integrated restriction enzyme sites.

To clone the flagellar major component encoding gene *fliC* [41] and the master regulator encoding genes *flhDC* [42] either singly or co-expressed in wt *Δasd Salmonella*, *fliC* and *flhDC* of *S*. Typhimurium strain H71 were amplified by polymerase chain reaction (PCR) with primers fliC-F+fliC-R and flhDC-F+flhDC-R, respectively (Table 1). Then *fliC* and *flhDC* were inserted into pV55 [43] to generate plasmids pTP2fliC and pTP2flhDC, respectively. To co-express *fliC* and *flhDC*, *fliC* from pTP2fliC was inserted into pTP2flhDC to construct plasmid pTP2DC+C. Thus, *fliC* in pTP2fliC, *flhDC* in pTP2flhDC, and both *fliC* and *flhDC* in pTP2DC+C are all under the control of the hybrid promoter of P*tetA*∼P*phoP* [43]. Plasmids pTP2fliC, pTP2flhDC, and pTP2DC+C were transformed to *S.* Typhimurium P1 to generate P1-pTP2fliC, -pTP2flhDC, and -pTP2DC+C, respectively. Strain P1-pY was used as a control [25].

### Western Blot Analysis

After overnight growth at 37°C on LB agar, P1-pTP2fliC, -pTP2flhDC, -pTP2DC+C, and -pY cells were harvested and flagella were sheared from cells by rigorous vortexing [44]. The mouse anti-FliC monoclonal Ab (clone 6H11, Santa Cruz Biotechnology, Santa Cruz, CA) was used as the primary Ab, and the goat anti-mouse IgG HRP-conjugated Ab was used as the secondary Ab (Southern Biotechnology Associates, Birmingham, AL). The procedure was as previously described [43], [45].

### Assessment of the Flagellated Salmonellae Viability

A previous study showed that water is able to exacerbate the autolysis of *E. coli* [46]. Thus, using Milli-Q water to treat flagellated salmonellae may reveal whether the membranes of the flagellated salmonellae are impaired. Sterile PBS, on the other hand, is an osmotically balanced buffer and is widely used as a vehicle for oral delivery [43]. Thus, sPBS was used as a control for Milli-Q water. Additionally, three temperatures, 4°C mimicking food (produce) chilling, room temperature (∼23°C) for gross maintenance [47], and 37°C, temperature mimicking normal human temperature, were used for the *ex vivo* viability assay.

After overnight growth on LB agar at 37°C, P1-pTP2fliC, -pTP2flhDC, -pTP2DC+C, and -pY cells were diluted in Milli-Q water or sPBS to a final concentration of ∼3 CFUs/µl. One-ml aliquots of each suspension were maintained at 4°C, 23°C, and 37°C, respectively, for five days. Each day, 10 µl of the cell suspension was plated onto LB agar, with six repeats (10 µl×6) per sample. Bacterial CFUs were enumerated, and the daily survival percentages were calculated in comparison to the corresponding initial populations on day 0.

### Evaluation of Infection and Replication of Flagellated Salmonellae in Macrophages

RAW264.7 macrophages (American Type Culture Collection) were used for evaluating the infection and replication of P1-pTP2fliC, -pTP2flhDC, -pTP2DC+C, and -pY. Infections, macrophage lysis, and bacterial CFU enumeration were conducted as previously described [25].

### Mouse Studies

Pathogen-free female BALB/c mice (National Cancer Institute, Frederick Cancer Research Facility) 7–9 weeks of age were used throughout this study. All mice were maintained at the Montana State University Animal Resource Center under pathogen-free conditions in individually ventilated cages under HEPA-filtered barrier conditions and were fed sterile food and water *ad libitum*. To assess the virulence of flagellated salmonellae, 1×10^9^ CFUs of P1-pTP2fliC, -pTP2flhDC, -pTP2DC+C, and -pY contained in 200 µl of sPBS were fed orally by gavage to BALB/c mice previously treated with a 100 µl 50% saturated sodium bicarbonate solution 30 min prior to challenge [48]. At four days post-infection, the spleens, Peyer’s patches, and livers were aseptically removed for determination of tissue weights and bacterial burden. Another group of mice dosed with the same protocol and procedure were monitored for survival for four weeks after infection, and the experiment was repeated twice. For challenge study, a dose of 5×10^7^ CFUs of wt H71 contained in 200 µl sPBS was given via oral gavage.

### Statistical Analysis

The Tukey Kramer multiple comparisons test was used for analyzing differences among experimental parameters. The Kaplan-Meier method (GraphPad Prism, GraphPad Software, Inc., La Jolla, CA) was utilized to obtain the mouse survival fractions after mice were administered with recombinant *Salmonella* strains. Using the Mantel-Haenszel log rank test, the *P*-values for statistical differences between attenuated *Salmonella* strains and control wt *Salmonella* strain-dosed mice were discerned at the 95% confidence interval. This method was also used for evaluating the protective efficacy after mice were challenged with wt *Salmonella* H71.
